# Predictive value of white blood cell to hemoglobin ratio for 30-day mortality in patients with severe intracerebral hemorrhage

**DOI:** 10.3389/fneur.2023.1222717

**Published:** 2024-01-12

**Authors:** Lei Liu, Xuetao Dong, Yaodong Liu, Shaozhen Wang, Liudong Wei, Lian Duan, Qingjun Zhang, Kun Zhang

**Affiliations:** Department of Neurosurgery, Chui Yang Liu Hospital Affiliated to Tsinghua University, Beijing, China

**Keywords:** white blood cell to hemoglobin ratio, mortality, intracerebral hemorrhage WHR for 30-day mortality of ICH, white blood cell count, hemoglobin

## Abstract

**Aim:**

To explore the predictive value of white blood cell to hemoglobin ratio (WHR) for 30-day mortality in patients with intracerebral hemorrhage (ICH).

**Methods:**

In this cohort study, 2,848 patients with ICH were identified in the Medical Information Mart for Intensive Care (MIMIC)-III and MIMIC-IV. Least absolute shrinkage and selection operator (LASSO) regression screened covariates of 30-day mortality of ICH patients. COX regression analysis was used to study the association of different levels of WHR, white blood cell (WBC), and hemoglobin (Hb) with 30-day mortality. The median follow-up time was 30 (20.28, 30.00) days.

**Results:**

In total, 2,068 participants survived at the end of the follow-up. WHR was negatively correlated with the Glasgow Coma Score (GCS) (spearman correlation coefficient = −0.143, *p* < 0.001), and positively associated with the Sepsis-related Organ Failure Assessment (SOFA) score (spearman correlation coefficient = 0.156, *p* < 0.001), quick SOFA (qSOFA) score (spearman correlation coefficient = 0.156, *p* < 0.001), and Simplified Acute Physiology Score II (SAPS-II) (spearman correlation coefficient = 0.213, *p* < 0.001). After adjusting for confounders, WHR >0.833 (HR = 1.64, 95%CI: 1.39–1.92) and WBC >10.9 K/uL (HR = 1.49, 95%CI: 1.28–1.73) were associated with increased risk of 30-day mortality of patients with ICH. The area under the curve (AUC) value of the prediction model based on WHR and other predictors was 0.78 (95%CI: 0.77–0.79), which was higher than SAPSII (AUC = 0.75, 95%CI: 0.74–0.76), SOFA score (AUC = 0.69, 95%CI: 0.68–0.70) and GCS (AUC = 0.59, 95%CI: 0.57–0.60).

**Conclusion:**

The level of WHR was associated with 30-day mortality in patients with severe ICH, and the WHR-based prediction model might provide a tool to quickly predict 30-day mortality in patients with ICH.

## Introduction

Intracerebral hemorrhage (ICH) is a devastating form of stroke, which accounts for 10–15% of all stroke cases (1). ICH results from a loss of vascular integrity leading to bleeding within the brain parenchyma, and the disease places a great burden on society (2, 3). Evidence has revealed that ICH is a significant cause of mortality and disability globally with in-hospital mortality of 32.4% and only 50% of patients having 1-year survivability (4, 5). The risk of mortality following an ICH is the highest in the acute phase, with 1-month fatality rates ranging from 13 to 61% (6). In China, the incidence of ICH was estimated at 11.9% (2.1 million cases) in participants aged 40 years and older (7). Despite advances in medical care, mortality and morbidity rates remain high, and effective treatment options are lacking. Identifying more reliable biomarkers for ICH is necessary for the improvement of the prognosis of these patients.

Hematoma in ICH was reported to trigger an inflammatory response, which promoted post-ICH brain injury and led to a poorer prognosis (8). Monitoring the early inflammatory response is of great significance for predicting the prognosis of patients with ICH. A variety of peripheral blood-based inflammatory markers, such as neutrophil-to-lymphocyte ratio (NLR), platelet-to-lymphocyte ratio (PLR), and the monocyte-to-high-density lipoprotein cholesterol ratio (MHR), were confirmed to be related to the near and long-term prognosis of patients with ICH or stroke patients (9–11). These studies mainly focused on specific blood cell subtypes. As an important part of the immune system, white blood cells (WBC) include various subtypes, such as neutrophils and lymphocytes, which may better reflect the changes in the systemic immune-inflammatory system (12). The absolute counts of WBC in the hematoma of patients with ICH tended to be greater at 12–30 h than they were within 12 h after ICH (13). A previous study documented that the incidence of anemia in hospitalized patients with ICH was as high as 72% (14). Low hemoglobin (Hb) levels were associated with poor prognosis in patients with ICH (15). To explore a biomarker combining WBC and Hb might be a more reliable index for the prognosis of ICH. WBC to Hb ratio (WHR), which evaluates the levels of WBC and Hb, was found to have better predictive value for postoperative survival of patients with gastric cancer than NLR and PLR (16). Given this, we speculated that WHR may be a more valuable predictor for the prognosis of patients with ICH. However, the predictive value of WHR for the prognosis of patients with ICH was still unclear.

In the present study, we aimed to explore the predictive value of WHR for 30-day mortality in patients with ICH based on the data from the Medical Information Mart for Intensive Care (MIMIC)-III and MIMIC-IV. We compared the predictive value of WHR for 30-day mortality of ICH patients with WBC or Hb. The prediction model for 30-day mortality of patients with ICH was constructed based on WHR, and the predictive performance was compared with the Simplified Acute Physiology Score II (SAPS-II) and Sepsis-related Organ Failure Assessment (SOFA) score. ICH samples from eICU Collaborative Research Database (eICU-CRD) were used as a validation set to verify the predictive value of the prediction model.

## Methods

### Study design and population

**Figure 1:**
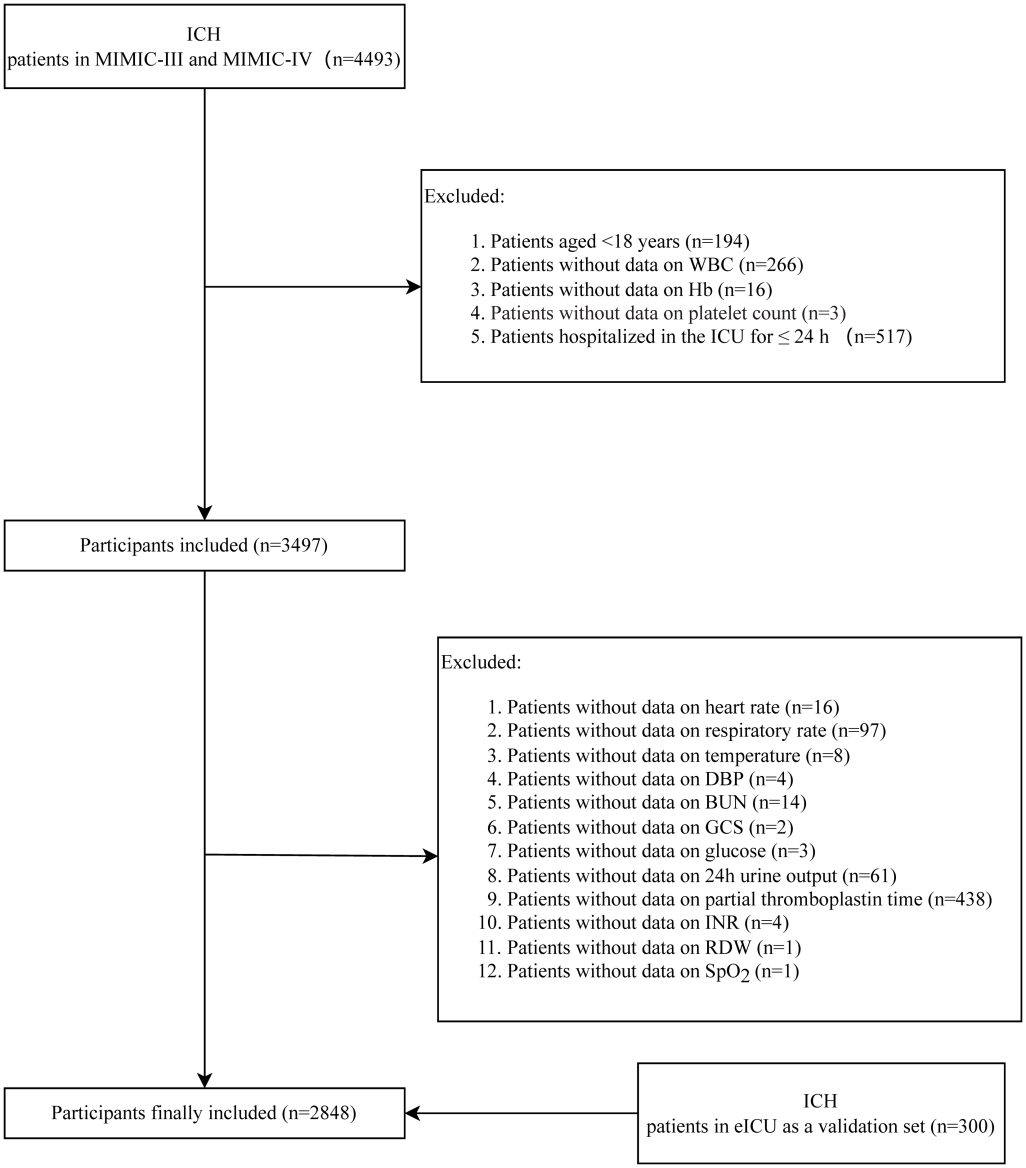
The screening process of the participants.

In this cohort study, 4,493 patients with ICH were identified in MIMIC-III and MIMIC-IV. MIMIC-III is a publicly available clinical database developed through a collaboration among the Massachusetts Institute of Technology, Philips Healthcare, and Beth Israel Deaconess Medical Center. This database includes information, such as vital signs, laboratory results, and medications, on patients admitted to various ICUs of the Beth Israel Deaconess Medical Center in Boston, Massachusetts, from 2001 to 2012 (17). MIMIC-IV, an update to MIMIC-III, incorporates contemporary data from 2012 to 2019 and improves on numerous aspects of MIMIC-III (18). The project was approved by the Institutional Review Boards of BIDMC (Boston, MA, United States) and the Massachusetts Institute of Technology (Cambridge, MA, United States). The requirement for individual patient consent was waived because the project did not impact clinical care and all protected health information was deidentified. In addition, the Ethics Review Committee of Chui Yang Liu Hospital affiliated with Tsinghua University has waived the requirement of informed consent for the study as the participants in this study were from a public database. In the current study, patients <18 years (*n* = 194), those without data on WBC (*n* = 266), Hb (*n* = 16), platelet count (*n* = 3), heart rate (*n* = 16), respiratory rate (*n* = 97), temperature (*n* = 8), diastolic blood pressure (DBP) (*n* = 4), blood urea nitrogen (BUN) (*n* = 14), Glasgow Coma Score (GCS) (*n* = 2), glucose (*n* = 3), 24 h urine output (*n* = 61), partial thromboplastin time (*n* = 438), International Normalized Ratio (INR) (*n* = 4), red cell distribution width (RDW) (*n* = 1), and oxygen saturation (SpO_2_) (*n* = 1), and participants who were hospitalized in the ICU ≤24 h (*n* = 517) were excluded. Finally, 2,848 patients with ICH were included. The screening process is exhibited in Figure 1. In addition, we extracted the data of 300 ICH patients >18 years from eICU-CRD as a validation set to verify the findings in the present study. The eICU-CRD is a multicenter database of over 200,000 ICU admissions in the United States (19).

### Potential covariates

Potential covariates included demographic variables [age (year), race (White, Black, other, or unknown), sex (female or male), insurance (Government, Medicaid, Medicare, Private, Self-Pay, or other)], treatment variables [mechanical ventilation (yes or no), vasopressors (yes or no), renal replacement therapy (yes or no), neurological dysfunction (yes or no), infectious diseases (yes or no), and surgery (yes or no)], and laboratory variables [heart rate (beat/min), systolic blood pressure (SBP, mmHg), DBP (mmHg), respiratory rate (beat/min), temperature (°C), SOFA score, quick SOFA (qSOFA) score, SAPSII, GCS, Charlson comorbidity index (CCI), platelet count (K/uL), RDW, hematocrit, creatinine (mg/dL), INR, prothrombin time (PT; sec), partial thromboplastin time (sec), BUN (mg/dL), glucose (mg/dL), bicarbonate (mEq/L), sodium (mEq/L), potassium (mEq/L), chloride (mEq/L), SpO_2_, and 24 h urine output (mL)]. All the data was analyzed using the data recorded during the first 24 h of ICU admission.

### Main and outcome variables

WHR was the main variable analyzed in the present study. WHR = WBC to Hb ratio was calculated using the following formula: (WBC [number/mm^3^])/(Hb [g/dL]).

WHR level, WBC level, and Hb level were categorized based on the best cutoff points using the surv_cutpoint() function of survminer in R (Institute for Statistics and Mathematics, Vienna, Austria). The best cutoff points were as follows: WHR of 0.833, WBC of 10.9 K /uL, and Hb of 11.1 g /dL.

Patients with ICH who survived or died within 30 days were outcomes in this study. The median follow-up time was 30 (20.28, 30.00) days.

### Statistical analysis

Normally distributed data were represented as mean ± standard deviation (SD) and a *t*-test was used for comparison between groups. Non-normally distributed data were presented as median and quartiles [M (Q_1_, Q_3_)]. Comparison between groups was performed by the Mann–Whitney U rank sum test. The categorical data were presented as the number of cases and percentages [n (%)], and the χ^2^-test was used for comparison between groups. Missing values were deleted, and sensitivity analysis was conducted on data before and after missing values deletion (Supplementary Table S1). The Spearman correlation coefficient was used to analyze the correlation between WHR and the severity of ICH (GCS, SOFA, QSOFA, and SAPSII). Least absolute shrinkage and selection operator (LASSO) regression screened covariates of 30-day mortality of patients with ICH, namely, age, heart rate, CCI, platelet count, RDW, PT, BUN, glucose, SpO_2_, 24 h urine output, mechanical ventilation, vasopressor, neurological dysfunction, and infectious diseases. The largest λ = 0.039 and log(λ) = −3.244 were selected, and predictors associated with the 30-day morality of patients with ICH were identified. COX regression analysis was used to study the associations of different levels of WHR, WBC, and Hb with 30-day mortality. Subgroup analysis was stratified by age (<65 years and ≥ 65 years), GCS (≥9 and < 9), SOFA (<3 and ≥ 3), and SAPSII (<33.5 and ≥ 33.5). Hazard ratio (HR) and 95% confidence interval (CI) were applied as effect size. ROC curves were plotted and AUC values were used to evaluate the predictive values of WHR for 30-day mortality of patients with ICH. Point estimation was applied to compare the differences between AUC values. SAS 9.4 (SAS Institute Inc., Cary, NC, United States) was employed for baseline data analysis and correlation analysis, and R version 4.2.1 (2022-06-23 ucrt) was applied for COX regression analysis and receiver operator characteristic (ROC) curve drawing. *p* < 0.05 was set as a statistical difference.

## Results

### Comparisons of baseline characteristics of patients with ICH who survived or died within 30 days

According to the results shown in Table 1, the median SOFA score (3.00 vs. 4.00) and SAPSII (31.00 vs. 36.00) in the WHR ≤ 0.833 group were lower than in the WHR >0.833 group. The mean RDW in the WHR ≤ 0.833 group was lower than in the WHR >0.833 group (14.22 vs. 14.40). The percentages of patients with neurological dysfunction (42.57% vs. 49.83%) and infectious diseases (30.67% vs. 37.79%) in the WHR ≤ 0.833 group were lower than in the WHR >0.833 group. The survival rate in the WHR ≤ 0.833 group was higher than in the WHR >0.833 group (81.01% vs. 65.02%). The detailed information of participants from eICU is shown in Supplementary Table S2.

**Table 1:** Comparisons of baseline characteristics of patients with ICH who survived or died within 30 days.

Variables	Total (*n* = 2,848)	WHR ≤0.833 (*n* = 1,353)	WHR >0.833 (*n* = 1,495)	Statistics	*P*
Age, year, Mean ± SD	66.87 ± 15.35	66.84 ± 15.27	66.91 ± 15.43	*t* = −0.13	0.895
Race, n (%)				χ^2^ = 13.552	0.004
White	1841 (64.64)	887 (65.56)	954 (63.81)		
Black	255 (8.95)	132 (9.76)	123 (8.23)		
Other	347 (12.18)	174 (12.86)	173 (11.57)		
Unknown	405 (14.22)	160 (11.83)	245 (16.39)		
Sex, n (%)				χ^2^ = 31.837	<0.001
Female	1,241 (43.57)	515 (38.06)	726 (48.56)		
Male	1,607 (56.43)	838 (61.94)	769 (51.44)		
Insurance, n (%)				χ^2^ = 8.738	0.120
Government	34 (1.19)	21 (1.55)	13 (0.87)		
Medicaid	192 (6.74)	96 (7.10)	96 (6.42)		
Medicare	1,407 (49.40)	644 (47.60)	763 (51.04)		
Other	865 (30.37)	433 (32.00)	432 (28.90)		
Private	330 (11.59)	152 (11.23)	178 (11.91)		
Self-Pay	20 (0.70)	7 (0.52)	13 (0.87)		
Mechanical ventilation, n (%)				χ^2^ = 116.161	<0.001
No	838 (29.42)	529 (39.10)	309 (20.67)		
Yes	2010 (70.58)	824 (60.90)	1,186 (79.33)		
Vasopressor, n (%)				χ^2^ = 80.740	<0.001
No	2,261 (79.39)	1,171 (86.55)	1,090 (72.91)		
Yes	587 (20.61)	182 (13.45)	405 (27.09)		
Renal replacement therapy, n (%)				χ^2^ = 14.714	<0.001
No	2,771 (97.30)	1,333 (98.52)	1,438 (96.19)		
Yes	77 (2.70)	20 (1.48)	57 (3.81)		
Surgery, n (%)				χ^2^ = 3.213	0.073
No	2,679 (94.07)	1,284 (94.90)	1,395 (93.31)		
Yes	169 (5.93)	69 (5.10)	100 (6.69)		
Neurological dysfunction, n (%)				χ^2^ = 15.056	<0.001
No	1,527 (53.62)	777 (57.43)	750 (50.17)		
Yes	1,321 (46.38)	576 (42.57)	745 (49.83)		
Infectious diseases, n (%)				χ^2^ = 15.953	<0.001
No	1868 (65.59)	938 (69.33)	930 (62.21)		
Yes	980 (34.41)	415 (30.67)	565 (37.79)		
Heart rate, bpm, Mean ± SD	81.90 ± 17.43	80.08 ± 16.53	83.55 ± 18.07	*t* = −5.35	<0.001
SBP, mmHg, Mean ± SD	139.19 ± 24.38	140.10 ± 24.21	138.36 ± 24.50	*t* = 1.91	0.056
DBP, mmHg, Mean ± SD	73.30 ± 17.49	75.08 ± 17.31	71.68 ± 17.50	*t* = 5.20	<0.001
Respiratory rate, bpm, Mean ± SD	18.21 ± 5.09	17.90 ± 4.98	18.49 ± 5.18	*t* = −3.10	0.002
Temperature, °C, Mean ± SD	36.79 ± 0.80	36.72 ± 0.72	36.86 ± 0.87	*t* = −4.59	<0.001
SOFA score, M (Q_1_, Q_3_)	3.00 (2.00, 5.00)	3.00 (2.00, 5.00)	4.00 (2.00, 6.00)	*Z* = −7.264	<0.001
QSOFA score, M (Q_1_, Q_3_)	2.00 (1.00, 2.00)	2.00 (1.00, 2.00)	2.00 (1.00, 3.00)	*Z* = −6.859	<0.001
SAPSII, M (Q_1_, Q_3_)	33.50 (26.00, 42.00)	31.00 (25.00, 38.00)	36.00 (28.00, 44.00)	*Z* = −10.023	<0.001
GCS, M (Q_1_, Q_3_)	13.00 (8.00, 14.00)	13.00 (10.00, 14.00)	12.00 (7.00, 15.00)	*Z* = 6.028	<0.001
CCI, M (Q_1_, Q_3_)	3.00 (1.00, 4.00)	3.00 (2.00, 4.00)	3.00 (1.00, 4.00)	*Z* = 0.295	0.768
WBC, K/uL, M (Q_1_, Q_3_)	10.10 (7.90, 13.10)	7.90 (6.40, 9.20)	12.90 (10.90, 15.70)	*Z* = −40.758	<0.001
Platelet, K/uL, M (Q_1_, Q_3_)	206.00 (160.00, 261.00)	186.00 (147.00, 236.00)	224.00 (177.00, 279.00)	*Z* = −12.639	<0.001
Hemoglobin, g/dL, Mean ± SD	11.87 ± 2.04	12.30 ± 1.98	11.49 ± 2.03	*t* = 10.80	<0.001
RDW, ratio, Mean ± SD	14.31 ± 1.75	14.22 ± 1.77	14.40 ± 1.72	*t* = −2.72	0.007
Hematocrit, ratio, Mean ± SD	35.29 ± 5.88	36.44 ± 5.80	34.26 ± 5.76	*t* = 10.04	<0.001
Creatinine blood, mg/dL, M (Q_1_, Q_3_)	0.90 (0.70, 1.10)	0.90 (0.70, 1.10)	0.90 (0.70, 1.20)	*Z* = −3.744	<0.001
INR, ratio, M (Q_1_, Q_3_)	1.20 (1.10, 1.30)	1.20 (1.10, 1.30)	1.20 (1.10, 1.30)	*Z* = −0.662	0.508
PT, sec, M (Q_1_, Q_3_)	13.00 (12.00, 14.40)	13.00 (11.90, 14.30)	13.10 (12.10, 14.40)	*Z* = −1.594	0.111
Partial thromboplastin time, sec, M (Q_1_, Q_3_)	27.60 (25.00, 30.80)	28.30 (25.60, 31.30)	27.00 (24.40, 30.10)	*Z* = 7.229	<0.001
BUN, mg/dL, M (Q_1_, Q_3_)	16.00 (12.00, 22.00)	16.00 (12.00, 20.00)	17.00 (12.00, 24.00)	*Z* = −6.075	<0.001
Glucose, mg/dL, M (Q_1_, Q_3_)	132.00 (111.00, 165.00)	124.00 (104.00, 150.00)	141.00 (118.00, 176.00)	*Z* = −11.948	<0.001
Bicarbonate, mEq/L, Mean ± SD	23.88 ± 3.72	24.41 ± 3.49	23.40 ± 3.85	*t* = 7.34	<0.001
Sodium, mEq/L, Mean ± SD	139.06 ± 4.84	139.15 ± 4.21	138.98 ± 5.35	*t* = 0.94	0.347
Potassium, mEq/L, Mean ± SD	3.94 ± 0.64	3.90 ± 0.58	3.97 ± 0.69	*t* = −2.54	0.011
Chloride, mEq/L, Mean ± SD	103.74 ± 5.51	103.68 ± 4.87	103.80 ± 6.04	*t* = −0.63	0.530
SpO_2_, ratio, Mean ± SD	97.84 ± 2.91	97.59 ± 2.99	98.07 ± 2.81	*t* = −4.39	<0.001
24 h urine output, mL, M (Q_1_, Q_3_)	1750.00 (1157.50, 2495.00)	1730.00 (1180.00, 2415.00)	1760.00 (1140.00, 2555.00)	*Z* = −0.466	0.641
WHR, ratio, M (Q_1_, Q_3_)	0.85 (0.66, 1.11)	0.64 (0.53, 0.75)	1.09 (0.94, 1.34)	*Z* = −46.151	<0.001
30-day prognosis, n (%)				χ^2^ = 91.295	<0.001
Survived	2068 (72.61)	1,096 (81.01)	972 (65.02)		
Dead	780 (27.39)	257 (18.99)	523 (34.98)		

SD, standard deviation; M, median; Q_1_, 1st quartile; Q_3_, 3st quartile; WHR, (white blood cell count [number/mm^3^])/(Hemoglobin level [g/dL]); ICH, intracerebral hemorrhage; SBP, systolic blood pressure; DBP, diastolic blood pressure; SOFA, Sepsis-related Organ Failure Assessment; qSOFA, quick Sepsis-related Organ Failure Assessment; SAPSII, Simplified Acute Physiology Score II; GCS, Glasgow Coma Score; CCI, Charlson comorbidity index; WBC, white blood cell; RDW, red cell distribution width; INR, international normalized ratio; PT, prothrombin time; BUN, blood urea nitrogen; SpO_2_, oxygen saturation.

### The association of WHR and the severity of ICH

The associations of WHR with GCS, SOFA score, qSOFA score, and SAPSII were evaluated via Spearman correlation analysis. The data depicted that WHR was negatively correlated with GCS (spearman correlation coefficient = −0.143, *p* < 0.001), and positively associated with SOFA score (spearman correlation coefficient = 0.156, *p* < 0.001), qSOFA score (spearman correlation coefficient = 0.156, *p* < 0.001), and SAPSII (spearman correlation coefficient = 0.213, *p* < 0.001) (Table 2). These results suggested that WHR was associated with the severity of ICH.

**Table 2:** The association of WHR and the severity of ICH.

Risk score system	Spearman correlation coefficient	*P*
GCS	−0.143	<0.001
SOFA	0.156	<0.001
QSOFA	0.156	<0.001
SAPSII	0.213	<0.001

WHR, (white blood cell count [number/mm^3^])/(Hemoglobin level [g/dL]); ICH, intracerebral hemorrhage; SOFA, Sepsis-related Organ Failure Assessment; qSOFA, quick Sepsis-related Organ Failure Assessment; SAPSII, Simplified Acute Physiology Score II.

### The associations of WHR, WBC, and Hb with the 30-day mortality of patients with ICH

**Figure 2:**
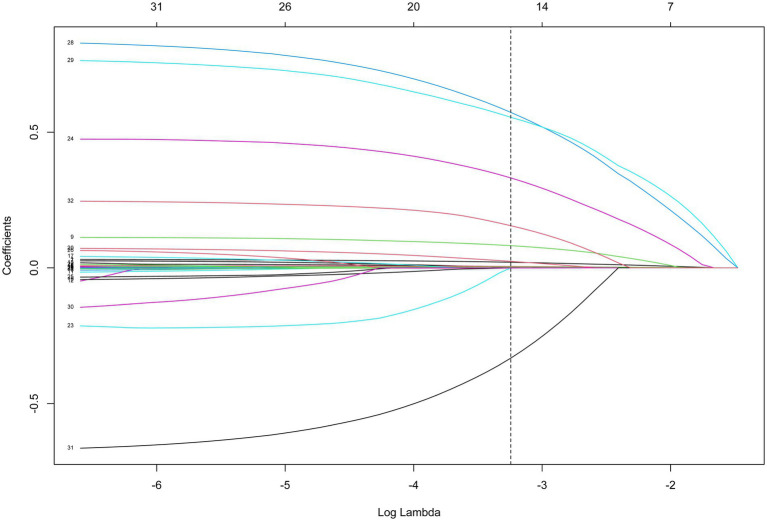
Lasso regression analysis identifying the covariates.

The potential confounding factors for 30-day mortality of patients with ICH were screened by LASSO regression analysis (Figure 2). The results indicated that age, heart rate, CCI, platelet count, RDW, PT, BUN, glucose, SpO_2_, 24 h urine output, mechanical ventilation, vasopressor, neurological dysfunction, and infectious diseases were confounders. According to the data displayed in Table 3, WHR >0.833 (HR = 2.07, 95%CI: 1.78–2.41) and WBC >10.9 K/uL (HR = 1.81, 95%CI: 1.57–2.08) might be correlated with elevated risk of 30-day mortality in patients with ICH. Hb >11.1 g/dL might be linked with reduced risk of 30-day mortality in patients with ICH (HR = 0.67, 95%CI: 0.58–0.77). After adjusting for age, heart rate, GCS, platelet count, RDW, PT, BUN, glucose, SpO_2_, 24 h urine output, mechanical ventilation, vasopressor, neurological dysfunction, and infectious diseases, WHR >0.833 (HR = 1.64, 95%CI: 1.39–1.92) and WBC >10.9 K/uL (HR = 1.49, 95%CI: 1.28–1.73) were associated with increased risk of 30-day mortality of patients with ICH (Table 3).

**Table 3:** The associations of WHR, WBC, and Hb with the 30-day mortality of patients with ICH.

Variable	Model 1		Model 2	
	HR (95%CI)	*P*	HR (95%CI)	*P*
WHR level				
≤0.833	Ref		Ref	
>0.833	2.07 (1.78–2.41)	<0.001	1.64 (1.39–1.92)	<0.001
WBC level				
≤10.9 K/uL	Ref		Ref	
>10.9 K/uL	1.81 (1.57–2.08)	<0.001	1.49 (1.28–1.73)	<0.001
Hb level				
≤11.1 g/dL	Ref		Ref	
>11.1 g/dL	0.67 (0.58–0.77)	<0.001	0.91 (0.78–1.06)	0.210

WHR, (white blood cell count [number/mm^3^])/(Hemoglobin level [g/dL]); WBC, white blood cell; Hb, Hemoglobin; Ref, reference; HR, hazard ratio; CI, confidence interval.

Model 1: unadjusted univariate COX regression model.

Model 2: Multivariable Cox regression model adjusting for age, heart rate, GCS, platelet count, RDW, PT, BUN, glucose, SpO_2_, 24 h urine output, mechanical ventilation, vasopressor, neurological dysfunction, and infectious diseases.

### Construction of the prediction model for 30-day mortality of patients with ICH based on WHR

**Figure 3:**
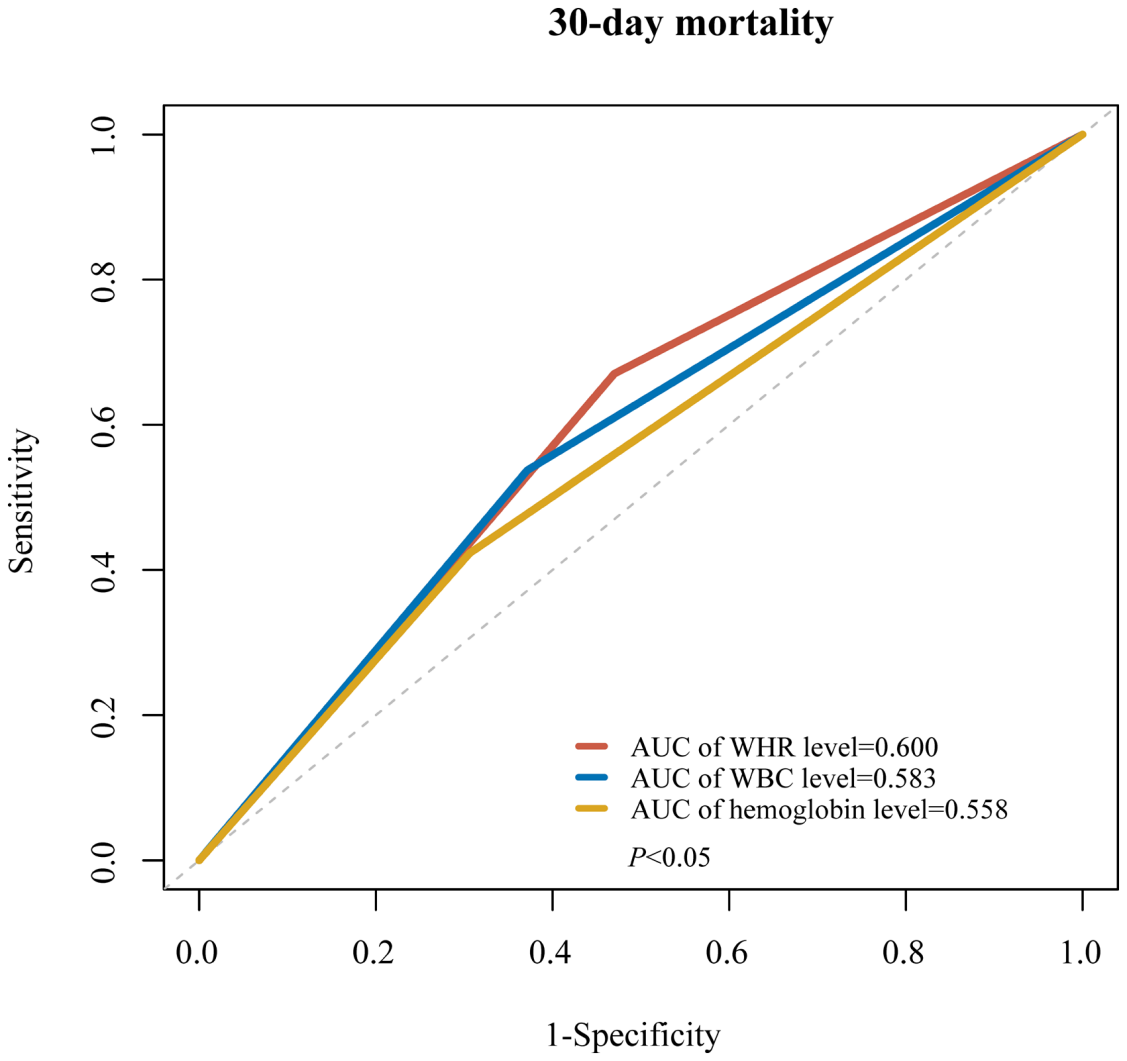
The AUC values of WHR, WBC, and Hb for 30-day mortality of patients with ICH.

The AUC values of WHR, WBC, and Hb for predicting the 30-day mortality of patients with ICH were 0.600, 0.583, and 0.558, respectively (Figure 3). The prediction model was established based on WHR, and variables, including age, heart rate, CCI, platelet count, RDW, PT, BUN, glucose, SpO_2_, 24 h urine output, mechanical ventilation, vasopressors, neurological dysfunction, and infectious diseases, correlated with 30-day mortality of patients with ICH. The AUC of the prediction model based on WHR and other predictors was 0.78 (95%CI: 0.77–0.79), which was higher than SAPSII (AUC = 0.75, 95%CI: 0.74–0.76), SOFA score (AUC = 0.69, 95%CI: 0.68–0.70), and GCS (AUC = 0.59, 95%CI: 0.57–0.60) in samples from MIMIC-III and MIMIC-IV according to point estimation (*p* < 0.05). Additionally, the AUC of the prediction model based on WHR and other predictors was higher than SAPSII (AUC = 0.60, 95%CI: 0.53–0.67), SOFA (AUC = 0.51, 95%CI: 0.45–0.58), and GCS (AUC = 0.58, 95%CI: 0.51–0.65). The AUC of the WHR-based prediction model was higher than SAPSII, SOFA, and GCS in samples from eICU (Table 4).

**Table 4:** Comparison of the predictive values of WHR with SAPSII and SOFA scores.

	MIMIC III and IV			eICU		
Variables	AUC	95%CI	*P*	AUC	95%CI	*P*
WHR^1^	0.78	0.77–0.79	Ref	0.73	0.65–0.82	Ref
SAPII	0.75	0.74–0.76	<0.001	0.60	0.53–0.67	0.021
SOFA	0.69	0.68–0.70	<0.001	0.51	0.45–0.58	<0.001
GCS	0.59	0.57–0.60	<0.001	0.58	0.51–0.65	0.007

WHR, (white blood cell count [number/mm^3^])/(Hemoglobin level [g/dL]); SOFA, Sepsis-related Organ Failure Assessment; SAPSII, Simplified Acute Physiology Score II; GCS, Glasgow Coma Score; CI, confidence interval; AUC, area under curve.

WHR^1^: Multivariable Cox regression model adjusting for age, heart rate, GCS, platelet count, RDW, PT, BUN, glucose, SpO_2_, 24 h urine output, mechanical ventilation, vasopressor, neurological dysfunction, and infectious diseases.

### Subgroup analysis of the associations of WHR, WBC, and Hb with the 30-day mortality of patients with ICH

In patients aged ≥65 years, WHR >0.833 and WBC >10.9 K/uL were associated with an increased risk of 30-day mortality of patients with ICH. WHR >0.833 and WBC >10.9 K/uL were correlated with elevated risk of 30-day mortality in both ICH patients with GCS <9 and GCS ≥ 9. And Hb >11.1 g/dL was associated with decreased risk of 30-day mortality of ICH patients with GCS ≥ 9. The increased risk of 30-day mortality of patients with ICH was observed in those with WHR >0.833, and WBC >10.9 K/uL in both the SOFA score < 3 group and the SOFA score ≥ 3 group. Hb >11.1 g/dL was associated with reduced risk of 30-day mortality of ICH patients with SOFA score ≥ 3. WHR >0.833 and WBC >10.9 K/uL were correlated with elevated risk of 30-day mortality in both ICH patients with SAPSII<33.5 and SAPSII≥33.5. WHR >0.833 and WBC >10.9 K/uL were correlated with an elevated risk of 30-day mortality in ICH patients with CCI <3, whereas WHR >0.833, WBC >10.9 K/Ul, and Hb >11.1 g/dL were linked to a lowered risk of 30-day mortality in ICH patients with CCI ≥3 (Table 5).

**Table 5:** Subgroup analysis of the associations of WHR, WBC, and Hb with the 30-day mortality of patients with ICH.

Variables	Age		GCS		SOFA		SAPSII		CCI	
	HR (95%CI)	*P*	HR (95%CI)	*P*	HR (95%CI)	*P*	HR (95%CI)	*P*	HR (95%CI)	*P*
	Age < 65		GCS < 9		SOFA<3		SAPSII<33.5		CCI < 3	
WHR level										
≤0.833	Ref		Ref		Ref		Ref		Ref	
>0.833	1.17 (0.88–1.55)	0.291	1.37 (1.08–1.75)	0.010	2.33 (1.55–3.51)	<0.001	1.98 (1.46–2.70)	<0.001	1.87 (1.42–2.46)	<0.001
WBC level										
≤10.9 K/uL	Ref		Ref		Ref		Ref		Ref	
>10.9 K/uL	1.01 (0.76–1.35)	0.922	1.32 (1.05–1.66)	0.016	1.99 (1.38–2.88)	<0.001	1.72 (1.27–2.33)	<0.001	1.87 (1.44–2.43)	<0.001
Hb level										
≤11.1 g/dL	Ref		Ref		Ref		Ref		Ref	
>11.1 g/dL	1.01 (0.74–1.37)	0.955	1.11 (0.88–1.40)	0.396	1.05 (0.72–1.52)	0.800	0.86 (0.62–1.20)	0.386	1.14 (0.88–1.49)	0.324
	Age ≥ 65		GCS ≥ 9		SOFA≥3		SAPSII≥33.5		CCI ≥ 3	
WHR level										
≤0.833	Ref		Ref		Ref		Ref		Ref	
>0.833	1.85 (1.53–2.25)	<0.001	1.63 (1.32–2.01)	<0.001	1.42 (1.19–1.69)	<0.001	1.39 (1.16–1.68)	<0.001	1.56 (1.28–1.89)	<0.001
WBC level										
≤10.9 K/uL	Ref		Ref		Ref		Ref		Ref	
>10.9 K/uL	1.72 (1.44–2.06)	<0.001	1.45 (1.18–1.78)	<0.001	1.33 (1.13–1.58)	<0.001	1.35 (1.13–1.61)	<0.001	1.34 (1.11–1.63)	0.003
Hb level										
≤11.1 g/dL	Ref		Ref		Ref		Ref		Ref	
>11.1 g/dL	0.93 (0.77–1.12)	0.439	0.80 (0.65–0.98)	0.034	0.93 (0.78–1.10)	0.413	1.03 (0.86–1.23)	0.742	0.77 (0.64–0.94)	0.009

WHR, (white blood cell count [number/mm^3^])/(Hemoglobin level [g/dL]); WBC, white blood cell; Hb, Hemoglobin; Ref, reference; HR, hazard ratio; CI, confidence interval; GCS, Glasgow Coma Score; SOFA, Sepsis-related Organ Failure Assessment; SAPSII, Simplified Acute Physiology Score II; CCI, Charlson comorbidity index; Ref, reference; HR, hazard ratio; CI, confidence interval; AUC, area under curve.

Multivariable Cox regression model, if not stratified, adjusting for age, heart rate, GCS, platelet count, RDW, PT, BUN, glucose, SpO_2_, 24 h urine output, mechanical ventilation, vasopressor, neurological dysfunction, and infectious diseases.

## Discussion

In the current study, the data of 4,493 patients with ICH were collected to evaluate the association between WHR and 30-day mortality of patients with ICH. The results delineated that a higher WHR level was associated with an increased risk of 30-day mortality of patients with ICH. WHR presented good predictive value for 30-day mortality of patients with ICH. The prediction model established based on WHR and other predictors had good predictive performance for 30-day mortality of patients with ICH. The finding might remind clinicians to frequently monitor the levels of WBC and Hb in patients with ICH, and provide a reliable biomarker for identifying ICH patients with a high risk of mortality within 30 days.

In a previous study, some scholars believed that WBC was a reliable predictor for new silent cerebral infarction after ICH (20). In several observational studies, high WBC level was reported to predict subsequent early neurological deterioration in patients with supratentorial ICH (21, 22). Another study demonstrated that increased WBC was indirectly associated with mortality or clinical outcome of acute ICH patients, and was more likely to reflect the extent of hematoma volume or clinical severity (23). There was evidence revealing that the severity of neurological damage after ICH was directly proportional to increased WBC count (24). ICH was independently correlated with elevated WBC count in patients with acute non-cerebral amyloid angiopathy (non-CAA) ICH (25). These findings support the results of our study, which depicted that a higher WBC level was associated with an elevated risk of 30-day mortality in patients with ICH. This may be because an elevated WBC count may reflect an inflammatory reaction related to the extent of initial brain injury in ICH (26). Another study suggested that reducing WBC counts after the onset of the disease can significantly improve the prognosis of patients with ICH (27).

On the other hand, the Hb level was also identified to be associated with the prognosis of patients with ICH. Acosta et al. found that higher Hb levels at admission were correlated with better functional outcomes in spontaneous ICH patients (15). Worse outcomes were identified in ICH patients with lower Hb levels (28, 29). Roh et al. indicated that lower Hb levels at admission were related to more hematoma expansion after ICH, and hematoma expansion was associated with worse outcomes after ICH (30). Herein, increased Hb was identified to be associated with a lower risk of 30-day mortality in patients with ICH in the unadjusted model. Notably, the incidence of anemia in samples from MIMIC-III and MIMIC-IV was 60.32%. Anemia may result from iron deficiency (31, 32), and might result in abnormal coagulation, hemostatic alterations, and an increased bleeding tendency (31, 32). Anemia might cause neuronal tissue hypoxia, metabolic distress, and cell energy dysfunction that possibly leads to pronounced secondary cerebral injury by reduced oxygen-carrying capacity (33). For patients with anemia, packed red blood cell transfusion might be considered (14). In addition, we identified higher WHR, which was based on WBC and Hb, was also associated with an elevated risk of 30-day mortality in patients with ICH. The predictive value of WHR for the risk of 30-day mortality in patients with ICH was better than WBC and Hb.

Currently, SOFA score and SAPSII contain the evaluation of multiple laboratory indicators, which are often used to predict the prognosis of diseases (34, 35). In the present study, a prediction model for 30-day mortality in patients with ICH was constructed based on WHR and other predictors. Compared with the traditional risk score systems, including the SOFA score and SAPSII, the predictive performance of the model based on WHR was better than the SOFA score and SAPSII. These data suggested that WHR might be a valuable predictor for 30-day mortality in patients with ICH. The findings of our study might provide a quick tool to identify ICH patients who have a high risk of death within 30 days, and clinicians should provide special care and treatments for these patients to improve their prognosis. WBC and Hb are easily obtained and could be widely applied in clinical settings. The recent consensus recommends a combination of initial treatment, such as the administration of steroids and IV immunoglobulins, along with platelet transfusion as an emergent intervention for patients experiencing active and significant bleeding. An individualized assessment to determine disease severity and prognosis stratification can greatly contribute to making informed therapeutic decisions. Our model might be valuable in guiding therapeutic options based on the estimated risk of mortality, encompassing specialized treatments, optimal timing of interventions, and a comprehensive assessment of treatment benefits and costs.

There were several limitations in the present study. First, due to the high missing values of 48 and 72 h WBC and Hb levels, the changes of WHR during follow-up were not analyzed. Second, some potential confounders might affect the WBC level and outcomes were not recorded in the database, and thus results could not be adjusted for these potential confounders. Third, MIMIC lacks image-related records, so hematoma volume and other prognostic indicators of ICH could not be obtained. GCS and other scores were used to reflect the severity of patients with ICH, and corresponding subgroup analysis was performed to verify the robustness of the association between WHR and the prognosis of patients with ICH. Fourth, due to the limitation of the MIMIC database, we could not calculate the ICH score in our participants and compare the predictive values between the ICH score and WHR. In the future, we will use data from our institution to compare the predictive values of ICH score and WHR, and therefore explore whether WHR combined with the ICH score can add the predictive accuracy for the prognosis of patients with ICH.

## Conclusion

This study collected data from 2,848 patients with ICH to assess the association between WHR and 30-day mortality of patients with ICH. The level of WHR was associated with 30-day mortality in patients with severe ICH and had a good predictive performance for 30-day mortality in patients with ICH. The findings might provide a reference for the importance of detecting WBC and Hb levels in patients with ICH, and offer a tool to early identify those patients with ICH who are at high risk of 30-day mortality, allowing for appropriate interventions to be applied.

## Data availability statement

The data analyzed in this study was obtained from the Medical Information Mart for Intensive Care (MIMIC)-III and MIMIC-IV databases, the following licenses/restrictions apply: To access the files, users must be credentialed users, complete the required training (CITI Data or Specimens Only Research) and sign the data use agreement for the project. Requests to access these datasets should be directed to MIMIC database, https://mimic.physionet.org/iii/ and https://mimic.physionet.org/iv/.

## Ethics statement

Ethical review and approval was not required for the study on human participants in accordance with the local legislation and institutional requirements. Written informed consent from the patients/participants or patients/participants' legal guardian/next of kin was not required to participate in this study in accordance with the national legislation and the institutional requirements.

## Author contributions

LL and KZ designed the study. LL wrote the manuscript. XD, YL, SW, LW, LD, and QZ collected, analyzed, and interpreted the data. KZ critically reviewed, edited, and approved the manuscript. All authors read and approved the final manuscript.
