# Supplementary material for: Predictive value of white blood cell to hemoglobin ratio for 30-day mortality in patients with severe intracerebral hemorrhage
Source: Front Neurol. 2024 Jan 12;14:1222717. doi: 10.3389/fneur.2023.1222717 (PMC10811233; doi:10.3389/fneur.2023.1222717)
Supplement: Supplementary file 1 [file Table_1.docx]

Supplementary Table 1 Sensitivity analysis of the data before and after missing values deletion

| Variables | Total (n=6345) | Before missing values deletion (n=3497) | After missing values deletion (n=2848) | Statistics | *P* |
| --- | --- | --- | --- | --- | --- |
| Age, year, Mean±SD | 66.99 ± 15.34 | 67.09 ± 15.33 | 66.87 ± 15.35 | t=0.55 | 0.582 |
| Race, n (%) |  |  |  | χ^2^=1.170 | 0.760 |
| White | 4131 (65.11) | 2290 (65.48) | 1841 (64.64) |  |  |
| Black | 572 (9.01) | 317 (9.06) | 255 (8.95) |  |  |
| Other | 772 (12.17) | 425 (12.15) | 347 (12.18) |  |  |
| Unknown | 870 (13.71) | 465 (13.30) | 405 (14.22) |  |  |
| Gender, n (%) |  |  |  | χ^2^=0.192 | 0.661 |
| Female | 2784 (43.88) | 1543 (44.12) | 1241 (43.57) |  |  |
| Male | 3561 (56.12) | 1954 (55.88) | 1607 (56.43) |  |  |
| Insurance, n (%) |  |  |  | χ^2^=0.983 | 0.964 |
| Government | 72 (1.13) | 38 (1.09) | 34 (1.19) |  |  |
| Medicaid | 419 (6.60) | 227 (6.49) | 192 (6.74) |  |  |
| Medicare | 3175 (50.04) | 1768 (50.56) | 1407 (49.40) |  |  |
| Other | 1911 (30.12) | 1046 (29.91) | 865 (30.37) |  |  |
| Private | 724 (11.41) | 394 (11.27) | 330 (11.59) |  |  |
| Self-Pay | 44 (0.69) | 24 (0.69) | 20 (0.70) |  |  |
| Mechanical ventilation, n (%) |  |  |  | χ^2^=1.130 | 0.288 |
| No | 1910 (30.10) | 1072 (30.65) | 838 (29.42) |  |  |
| Yes | 4435 (69.90) | 2425 (69.35) | 2010 (70.58) |  |  |
| Vasopressors, n (%) |  |  |  | χ^2^=0.223 | 0.637 |
| No | 5054 (79.65) | 2793 (79.87) | 2261 (79.39) |  |  |
| Yes | 1291 (20.35) | 704 (20.13) | 587 (20.61) |  |  |
| Renal replacement therapy, n (%) |  |  |  | χ^2^=0.821 | 0.365 |
| No | 6160 (97.08) | 3389 (96.91) | 2771 (97.30) |  |  |
| Yes | 185 (2.92) | 108 (3.09) | 77 (2.70) |  |  |
| Surgery, n (%) |  |  |  | χ^2^=0.348 | 0.555 |
| No | 5956 (93.87) | 3277 (93.71) | 2679 (94.07) |  |  |
| Yes | 389 (6.13) | 220 (6.29) | 169 (5.93) |  |  |
| Heart rate, bpm, Mean±SD | 81.88 ± 17.38 | 81.86 ± 17.33 | 81.90 ± 17.43 | t=-0.10 | 0.921 |
| Systolic, mmHg, Mean±SD | 139.36 ± 24.40 | 139.50 ± 24.43 | 139.19 ± 24.38 | t=0.52 | 0.606 |
| Diastolic, mmHg, Mean±SD | 73.34 ± 17.51 | 73.38 ± 17.53 | 73.30 ± 17.49 | t=0.18 | 0.861 |
| Respiratory rate, bpm, Mean±SD | 18.20 ± 5.08 | 18.20 ± 5.07 | 18.21 ± 5.09 | t=-0.05 | 0.960 |
| Temperature, ℃, Mean±SD | 36.79 ± 0.79 | 36.79 ± 0.79 | 36.79 ± 0.80 | t=-0.04 | 0.971 |
| SOFA, M (Q_1_, Q_3_) | 3.00 (2.00, 5.00) | 4.00 (2.00, 5.00) | 3.00 (2.00, 5.00) | Z=0.126 | 0.900 |
| QSOFA, M (Q_1_, Q_3_) | 2.00 (1.00, 2.00) | 2.00 (1.00, 2.00) | 2.00 (1.00, 2.00) | Z=0.878 | 0.380 |
| SAPSII, M (Q_1_, Q_3_) | 33.00 (26.00, 42.00) | 33.00 (26.00, 41.00) | 33.50 (26.00, 42.00) | Z=0.804 | 0.421 |
| GCS, M (Q_1_, Q_3_) | 13.00 (8.00, 14.00) | 13.00 (8.00, 14.00) | 13.00 (8.00, 14.00) | Z=0.255 | 0.798 |
| CCI, M (Q_1_, Q_3_) | 3.00 (1.00, 4.00) | 3.00 (1.00, 4.00) | 3.00 (1.00, 4.00) | Z=-0.102 | 0.919 |
| WBC, K/uL, M (Q_1_, Q_3_) | 10.10 (7.90, 13.10) | 10.20 (7.90, 13.10) | 10.10 (7.90, 13.10) | Z=-0.290 | 0.772 |
| Platelet, K/uL, M (Q_1_, Q_3_) | 208.00 (161.00, 262.00) | 209.00 (161.00, 263.00) | 206.00 (160.00, 261.00) | Z=-0.839 | 0.401 |
| Hemoglobin, g/dL, Mean ± SD | 11.89 ± 2.04 | 11.90 ± 2.04 | 11.87 ± 2.04 | t=0.50 | 0.614 |
| RDW, ratio, Mean ± SD | 14.32 ± 1.74 | 14.32 ± 1.74 | 14.31 ± 1.75 | t=0.29 | 0.772 |
| Hematocrit, ratio, Mean ± SD | 35.33 ± 5.87 | 35.36 ± 5.86 | 35.29 ± 5.88 | t=0.46 | 0.646 |
| Creatinine blood, mg/dL, M (Q_1_, Q_3_) | 0.90 (0.70, 1.10) | 0.90 (0.70, 1.10) | 0.90 (0.70, 1.10) | Z=-0.567 | 0.571 |
| INR, ratio, M (Q_1_, Q_3_) | 1.20 (1.10, 1.30) | 1.20 (1.10, 1.30) | 1.20 (1.10, 1.30) | Z=-0.211 | 0.833 |
| PT, sec, M (Q_1_, Q_3_) | 13.00 (12.00, 14.40) | 13.00 (12.00, 14.40) | 13.00 (12.00, 14.40) | Z=-0.247 | 0.805 |
| Partial thromboplastin time, sec, M (Q_1_, Q_3_) | 27.70 (25.00, 30.90) | 27.70 (25.00, 30.90) | 27.60 (25.00, 30.80) | Z=-0.720 | 0.472 |
| BUN, mg/dL, M (Q_1_, Q_3_) | 16.00 (12.00, 22.00) | 16.00 (12.00, 22.00) | 16.00 (12.00, 22.00) | Z=-0.452 | 0.651 |
| Glucose, mg/dL, M (Q_1_, Q_3_) | 132.00 (110.00, 165.00) | 132.00 (110.00, 164.00) | 132.00 (111.00, 165.00) | Z=0.489 | 0.625 |
| Bicarbonate, mEq/L, Mean ± SD | 23.90 ± 3.73 | 23.92 ± 3.74 | 23.88 ± 3.72 | t=0.50 | 0.617 |
| Sodium, mEq/L, Mean ± SD | 139.04 ± 4.82 | 139.03 ± 4.80 | 139.06 ± 4.84 | t=-0.28 | 0.778 |
| Potassium, mEq/L, Mean ± SD | 3.94 ± 0.64 | 3.94 ± 0.65 | 3.94 ± 0.64 | t=0.36 | 0.722 |
| Chloride, mEq/L, Mean ± SD | 103.73 ± 5.49 | 103.72 ± 5.47 | 103.74 ± 5.51 | t=-0.15 | 0.880 |
| SpO_2_, ratio, Mean ± SD | 97.79 ± 3.14 | 97.75 ± 3.33 | 97.84 ± 2.91 | t=-1.12 | 0.264 |
| 24 h urine output, mL, M (Q_1_, Q_3_) | 1745.00 (1150.00, 2475.00) | 1740.00 (1145.00, 2462.50) | 1750.00 (1157.50, 2495.00) | Z=0.513 | 0.608 |
| WHR, ratio, M (Q_1_, Q_3_) | 0.86 (0.65, 1.12) | 0.86 (0.65, 1.12) | 0.85 (0.66, 1.11) | Z=-0.182 | 0.855 |
| Outcome, n (%) |  |  |  | χ^2^=0.009 | 0.924 |
| Survival | 4611 (72.67) | 2543 (72.72) | 2068 (72.61) |  |  |
| Death | 1734 (27.33) | 954 (27.28) | 780 (27.39) |  |  |

SD: standard deviation, M: median, Q_1_:1st quartile, Q_3_:3st quartile, WHR: white blood cell count [number/mm^3^])/(Hemoglobin level [g/dL]), ICH: intracerebral hemorrhage, SBP: systolic blood pressure, DBP: diastolic blood pressure, SOFA: Sepsis-related Organ Failure Assessment, qSOFA: quick Sepsis-related Organ Failure Assessment, SAPSII: Simplified Acute Physiology Score II, GCS: Glasgow Coma Score, CCI: Charlson comorbidity index, WBC: white blood cell, RDW: red cell distribution width, INR: international normalized ratio, PT: prothrombin time, BUN: blood urea nitrogen, SpO_2_: oxygen saturation

Supplementary Table 2 Comparisons of baseline characteristics of ICH patients from eICU who survived or died within 30 days

|  | | WHR level | |  | |
| --- | --- | --- | --- | --- | --- |
| Variables | Total (n=300) | ≤0.833 (n=193) | >0.833 (n=107) | Statistics | *P* |
| Age, year, Mean±SD | 64.30 ± 15.27 | 64.94 ± 14.47 | 63.16 ± 16.63 | t=0.97 | 0.335 |
| Race, n (%) |  |  |  | - | 0.193 |
| African American | 61 (20.33) | 40 (20.73) | 21 (19.63) |  |  |
| Asian | 8 (2.67) | 7 (3.63) | 1 (0.93) |  |  |
| Caucasian | 189 (63.00) | 115 (59.59) | 74 (69.16) |  |  |
| Hispanic | 17 (5.67) | 12 (6.22) | 5 (4.67) |  |  |
| Native American | 3 (1.00) | 1 (0.52) | 2 (1.87) |  |  |
| Other/Unknown | 22 (7.33) | 18 (9.33) | 4 (3.74) |  |  |
| Gender, n (%) |  |  |  | χ^2^=3.540 | 0.060 |
| Female | 138 (46.00) | 81 (41.97) | 57 (53.27) |  |  |
| Male | 162 (54.00) | 112 (58.03) | 50 (46.73) |  |  |
| Mechanical ventilation, n (%) |  |  |  | χ^2^=7.252 | 0.007 |
| No | 190 (63.33) | 133 (68.91) | 57 (53.27) |  |  |
| Yes | 110 (36.67) | 60 (31.09) | 50 (46.73) |  |  |
| Vasopressors, n (%) |  |  |  | χ^2^=1.919 | 0.166 |
| No | 280 (93.33) | 183 (94.82) | 97 (90.65) |  |  |
| Yes | 20 (6.67) | 10 (5.18) | 10 (9.35) |  |  |
| Renal replacement therapy, n (%) |  |  |  | - | 0.462 |
| No | 292 (97.33) | 189 (97.93) | 103 (96.26) |  |  |
| Yes | 8 (2.67) | 4 (2.07) | 4 (3.74) |  |  |
| Surgery, n (%) |  |  |  | χ^2^=3.688 | 0.055 |
| No | 278 (92.67) | 183 (94.82) | 95 (88.79) |  |  |
| Yes | 22 (7.33) | 10 (5.18) | 12 (11.21) |  |  |
| Neurological dysfunction, n (%) |  |  |  | χ^2^=4.062 | 0.044 |
| No | 212 (70.67) | 144 (74.61) | 68 (63.55) |  |  |
| Yes | 88 (29.33) | 49 (25.39) | 39 (36.45) |  |  |
| Infectious diseases, n (%) |  |  |  | χ^2^=0.087 | 0.768 |
| No | 282 (94.00) | 182 (94.30) | 100 (93.46) |  |  |
| Yes | 18 (6.00) | 11 (5.70) | 7 (6.54) |  |  |
| Heart rate, bpm, Mean±SD | 84.55 ± 18.28 | 83.26 ± 17.26 | 86.86 ± 19.86 | t=-1.64 | 0.103 |
| Systolic, mmHg, Mean±SD | 162.01 ± 29.98 | 164.75 ± 29.19 | 157.20 ± 30.88 | t=2.04 | 0.042 |
| Diastolic, mmHg, Mean±SD | 86.76 ± 22.88 | 88.83 ± 23.34 | 83.04 ± 21.65 | t=2.07 | 0.039 |
| Respiratory rate, bpm, Mean±SD | 18.81 ± 4.17 | 18.46 ± 3.84 | 19.43 ± 4.65 | t=-1.79 | 0.075 |
| Temperature, ℃, Mean±SD | 36.79 ± 0.62 | 36.73 ± 0.61 | 36.89 ± 0.64 | t=-2.07 | 0.039 |
| SOFA, score, M (Q_1_, Q_3_) | 4.00 (1.00, 6.00) | 4.00 (1.00, 5.00) | 4.00 (2.00, 8.00) | Z=2.128 | 0.033 |
| QSOFA, score, M (Q_1_, Q_3_) | 2.00 (1.00, 2.00) | 1.00 (1.00, 2.00) | 2.00 (1.00, 2.00) | Z=1.647 | 0.100 |
| SAPSII, score, M (Q_1_, Q_3_) | 25.00 (16.00, 37.00) | 22.00 (15.00, 34.00) | 29.00 (21.00, 40.00) | Z=3.400 | <0.001 |
| Glasgow Coma Scale (GCS), M M (Q_1_, Q_3_) | 11.00 (6.00, 14.00) | 12.00 (6.00, 14.00) | 8.00 (4.00, 13.00) | Z=-2.480 | 0.013 |
| Charlson comorbidity index, M (Q_1_, Q_3_) | 4.00 (3.00, 5.00) | 4.00 (3.00, 5.00) | 4.00 (3.00, 5.00) | Z=0.357 | 0.721 |
| WBC, K/uL, M (Q_1_, Q_3_) | 9.60 (7.40, 12.51) | 7.90 (6.40, 9.70) | 14.10 (12.10, 16.30) | Z=12.716 | <0.001 |
| Platelet count, K/uL, M (Q_1_, Q_3_) | 214.00 (176.50, 263.00) | 208.00 (170.00, 248.00) | 235.00 (195.00, 284.00) | Z=3.563 | <0.001 |
| Hemoglobin, g/dL, Mean±SD | 13.39 ± 2.09 | 13.83 ± 1.87 | 12.59 ± 2.25 | t=4.83 | <0.001 |
| RDW, ratio, Mean±SD | 14.32 ± 1.91 | 14.10 ± 1.89 | 14.73 ± 1.88 | t=-2.80 | 0.005 |
| Hematocrit, ratio, Mean±SD | 40.01 ± 5.77 | 41.16 ± 5.12 | 37.93 ± 6.30 | t=4.55 | <0.001 |
| Creatinine, mg/dL, M (Q_1_, Q_3_) | 0.93 (0.73, 1.24) | 0.91 (0.75, 1.17) | 0.97 (0.72, 1.43) | Z=1.225 | 0.220 |
| INR, ratio, M (Q_1_, Q_3_) | 1.00 (1.00, 1.13) | 1.00 (1.00, 1.10) | 1.10 (1.00, 1.20) | Z=2.627 | 0.009 |
| PT, sec, M (Q_1_, Q_3_) | 12.90 (11.45, 14.00) | 12.90 (11.40, 13.70) | 13.10 (11.50, 14.40) | Z=1.668 | 0.095 |
| Partial thromboplastin time, sec, M (Q_1_, Q_3_) | 28.18 ± 5.36 | 28.27 ± 5.60 | 27.98 ± 4.86 | t=0.35 | 0.726 |
| BUN, mg/dL, M (Q_1_, Q_3_) | 16.00 (13.00, 22.50) | 16.00 (13.00, 20.00) | 18.00 (13.00, 27.00) | Z=2.368 | 0.018 |
| Glucose, mg/dL, M (Q_1_, Q_3_) | 130.50 (108.50, 163.00) | 126.00 (106.00, 153.00) | 141.00 (117.00, 180.00) | Z=3.165 | 0.002 |
| Bicarbonate, mEq/L, Mean±SD | 24.93 ± 3.21 | 25.26 ± 3.17 | 24.32 ± 3.22 | t=2.43 | 0.016 |
| Sodium, mEq/L, Mean±SD | 138.63 ± 3.92 | 138.87 ± 3.40 | 138.20 ± 4.70 | t=1.31 | 0.193 |
| Potassium, mEq/L, Mean±SD | 3.84 ± 0.60 | 3.79 ± 0.53 | 3.94 ± 0.71 | t=-1.90 | 0.059 |
| Chloride, mEq/L, Mean±SD | 103.15 ± 4.71 | 103.56 ± 4.35 | 102.40 ± 5.24 | t=1.95 | 0.052 |
| SPO2, ratio, Mean±SD | 95.76 ± 8.90 | 95.77 ± 9.30 | 95.76 ± 8.16 | t=0.01 | 0.995 |
| 24h urine output, mL, M (Q_1_, Q_3_) | 1625.00 (911.00, 2331.50) | 1470.00 (900.00, 2175.00) | 1756.00 (967.00, 2625.00) | Z=2.032 | 0.042 |
| WHR, ratio, M (Q_1_, Q_3_) | 0.73 (0.53, 0.97) | 0.59 (0.47, 0.70) | 1.10 (0.92, 1.27) | Z=14.346 | <0.001 |
| 30-day prognosis, n (%) |  |  |  | χ^2^=4.222 | 0.040 |
| Survived | 248 (82.67) | 166 (86.01) | 82 (76.64) |  |  |
| Dead | 52 (17.33) | 27 (13.99) | 25 (23.36) |  |  |

SD: standard deviation, M: median, Q1:1st quartile, Q3:3st quartile, WHR: white blood cell count [number/mm^3^])/(Hemoglobin level [g/dL]), ICH: intracerebral hemorrhage, SBP: systolic blood pressure, DBP: diastolic blood pressure, SOFA: Sepsis-related Organ Failure Assessment, qSOFA: quick Sepsis-related Organ Failure Assessment, SAPSII: Simplified Acute Physiology Score II, GCS: Glasgow Coma Score, CCI: Charlson comorbidity index, WBC: white blood cell, RDW: red cell distribution width, INR: international normalized ratio, PT: prothrombin time, BUN: blood urea nitrogen, SpO2: oxygen saturation
